# Supplementary material for: Are carbon emissions trading and green financial instruments synergistic? -Comprehensive quantitative research based on content analysis
Source: PLoS One. 2024 Mar 7;19(3):e0298601. doi: 10.1371/journal.pone.0298601 (PMC10919601; doi:10.1371/journal.pone.0298601)
Supplement: S1 Data — (ZIP) [file pone.0298601.s001.zip › Data/地级市Z_F工作报告.zip!/║╙─╧/┬σ╤⌠╩╨/└┤╫╘╣1⁄2╓┌║┼í╛┬φ┐╦╩2╛▌═°í┐_2019.docx]

各位代表：

现在，我代表市人民政府向大会作工作报告，请予审议，并请各位政协委员和列席人士提出意见。

一、2018年工作回顾

2018年是我市发展进程中极不平凡的一年。在省委、省政府和市委的正确领导下，在市人大、市政协的监督支持下，市政府与全市人民一道，高举习近平新时代中国特色社会主义思想伟大旗帜，全面贯彻党的十九大和十九届二中、三中全会精神，深入贯彻习近平总书记视察指导河南时的重要讲话精神，认真落实党中央国务院、省委省政府和市委决策部署，坚持稳中求进工作总基调，统筹推进“五位一体”总体布局，协调推进“四个全面”战略布局，全面深化“9+2”工作布局，着力推动“六个转型”，经济社会发展实现了稳中有进、稳中向好，较好地完成了市第十五届人大一次会议确定的目标任务。2018年全市地区生产总值达到4640.8亿元，同比增长7.9%，增速高于全国、全省平均水平。

——经济结构更趋优化。“三产超二产”的格局进一步巩固，服务业增速高于生产总值增速1.1个百分点，占GDP的比重同比提高2个百分点，三次产业结构调整为5.1︰44.6︰50.3。“消费超投资”的态势逐步显现，社会消费品零售总额同比增长10.3%，高于固定资产投资增速0.3个百分点。“新兴超传统”的基础不断夯实，六大高成长性制造业和高新技术产业对全市规上工业增长的贡献率分别达到39.1%和48.6%。

——质量效益持续提升。洛阳石化、中信重工、一拖集团等9家企业主营业务收入超百亿元，形成了装备制造、新材料、机器人及智能制造3个千亿级产业集群。15家市级试点企业盈利能力明显提升，50户重点企业实现产值增长11.7%，带动全市规上工业利润增长31.5%，高于全省7个百分点。万元规模以上工业增加值能耗下降10.47%，降幅大于全省平均水平。一般公共预算收入达到342.7亿元、增长5.1%。居民人均可支配收入24882元、增长9%。

——三大攻坚成效显著。积极防范化解金融、安全等重点领域风险，守住了不发生系统性区域性风险的底线。脱贫攻坚步伐坚实，实现11.74万贫困人口脱贫，栾川、洛宁、宜阳、伊川4县有望脱贫摘帽。污染防治攻坚强力推进，PM10、PM2.5、优良天数实现“两降一升”，水环境质量总体达标，我市入选国家清洁取暖试点城市，通过全国水生态文明城市建设试点验收。

——创新资源加快汇聚。自创区“一核四区多点”联动发展格局初步形成，自贸区入驻企业突破万家，规模500亿元的省军民融合产业投资基金成功落地。全市新增创新主体2228个、创新平台（载体）346个（其中国家级8个），分别是年度目标的2倍和1.2倍；国家高新技术企业、科技型中小企业分别达到470家、3125家。连续三年成功举办中国创新创业大赛先进制造行业总决赛，“2018聚力产业创新院士洛阳行”活动达成实质性合作项目49个。

——大事要事捷报频传。国家重要交通枢纽建设迈出坚实步伐，呼南高铁豫西通道南北节点接轨方案获得中国铁路总公司批复，动车组存车场开工建设，三洋铁路、蒙华铁路洛阳段快速推进，尧栾西、洛济等6条高速公路同时在建，“三横三纵三环”的高速路网加快形成。六大产业园建设提速发力，洛阳石化炼油扩能改造、格力智能制造、银隆新能源汽车等一批事关长远的重大项目加快实施。我市获得国家拟通报表彰的老工业基地调整改造真抓实干成效明显城市提名。

——城市建设亮点纷呈。古城快速路、新伊大街等建成通车，轨道交通、王城大道快速路等重点工程加快实施，形象靓丽、功能完善的市民之家、市规划展示馆成为城市新名片，隋唐洛阳城国家历史文化公园、二里头遗址博物馆加快建设，69家博物馆、106座城市书房交相辉映，兴洛湖公园、伊水游园、文博体育公园生态良好、环境优美，150个城市游园星罗棋布、绿意盎然，古今辉映、山环水润的城市特色日益彰显。全省百城建设提质工程和文明城市创建工作推进会在洛成功召开，洛阳“颜值”和“气质”被广泛点赞。

一年来，我们主要做了以下工作：

（一）着力稳企业、促增长，经济运行稳中有进。牢固树立服务企业的“店小二”理念，以地企合作为平台，以项目建设为抓手，切实加强经济运行研判调度，真心实意帮助企业解决困难，促进实体经济持续健康发展。深化地企合作。4次联席会议签约产业项目240个、总投资744亿元，项目开工率85%；238个产销对接合同资金履约率92.1%；70个科技成果项目转化实施65个；184个产融合作协议履约资金253.7亿元。减轻企业负担。办理企业减免税104.4亿元，出口退税10.8亿元。发放稳岗补贴4627万元，惠及415家企业1.3万人。降低企业社保费率，减轻企业、职工负担7.87亿元。推进大用户直购电交易，降低一般工商业企业电价。持续开展还贷周转金业务，节约企业融资成本2.1亿元。加强要素保障。155家企业通过资本市场融资275亿元。银企洽谈会签约资金1007.1亿元，落实率97.9%；人民币各项贷款余额增长17.2%，余额存贷比70%、同比提高4.6个百分点。加强生产要素应急调度，稳定煤电油气运供应。深化土地利用集中整治，重大项目用地需求得到保障。扩大有效投资。滚动实施合理有效投资行动计划，433个省市重点项目完成投资1472.9亿元，是年度计划的1.4倍。投资结构不断优化，高技术产业、基础设施、科技服务业投资分别增长27.8%、35.5%和72.7%。壮大民营经济。出台实施优化营商环境三年行动方案和促进民营经济高质量发展的具体意见，入选省优秀非公有制企业6家，东大科技产业园入选国家中小企业公共服务示范平台。预计全年非公有制经济增加值达到2858.7亿元，同比增长8.2%。

（二）着力调结构、促转型，产业升级步伐加快。坚持“四双联动”，强化创新引领，大力实施20个重点攻坚产业行动计划，推动一批重大专项项目落地见效，加快构建“565”现代产业体系。创新成果加速转化应用。实施市级以上重大科技专项76项，预计新增产值105亿元。12个创新引领型产业集群专项课题获省立项支持，工业CT项目加快推进，时速250公里的高铁轴承研制任务顺利完成，国内首台纯电动无人驾驶拖拉机田间试验成功。向231家企业发放科技创新券1.6亿元，直接引导企业研发投入12.3亿元。新建省院士工作站4个、首批中原学者科学家工作室3个。选树“河洛大工匠”10名、“河洛工匠”65名。清华大学博清科技、大连理工大学星派科技等40余个优质创业项目顺利落户。科技金融大市场挂牌科技成果529项，技术合同成交额36.5亿元。万人发明专利拥有量同比增长10.5%。传统产业加快改造提升。实施“三大改造”重点项目296个，完成投资263.8亿元。企业技术改造投资增长39.3%；麦斯克电子智能制造项目入选全国试点，中信重工入选国家级服务型制造企业，15家企业入选省级智能工厂（车间）；双瑞万基钛业等3家企业和龙鼎铝业分别入选国家级、省级绿色工厂。实施智能制造和工业互联网发展三年行动计划，机器人及智能装备产业主营业务收入增长25%，29个产品入选省首台（套）重大技术装备，20个项目入选省机器人“十百千”示范应用倍增工程。组建市工业互联网联盟，培育建设工业互联网云平台6个。中信重工、一拖集团工业互联网平台入选国家工业互联网试点示范，4家企业入选省制造业与互联网融合发展试点示范。新兴产业快速成长壮大。中原大数据云计算中心一期等82个项目顺利竣工，中储综合物流产业园、华晟物流园建设稳步推进。互联网及相关服务业营业收入增长49.4%，新能源、电子信息产业主营业务收入分别增长23.9%和21.7%。电商交易额突破2000亿元，快递企业业务量突破1亿件。文化旅游业加速融合。隋唐洛阳城应天门、两坊一街、南城墙等工程加快实施，第36届中国洛阳牡丹文化节、首届世界古都论坛、2018河洛文化旅游节成功举办，全年接待游客1.32亿人次，旅游总收入1145亿元，分别增长6.2%和10%。现代农业加快发展。粮食总产达50.1亿斤。发展特色种植176万亩。23个沟域经济示范区实施项目205个。市级以上龙头企业达到352个，新增省级淘宝镇1个、淘宝村8个。创建A级乡村旅游景区22家。我市入选全国新型职业农民培育示范市。

（三）着力促改革、扩开放，动力活力不断迸发。坚定不移全面深化改革，加快发展开放型经济，发展动能进一步增强。重点改革深入推进。持续深化供给侧结构性改革，提前完成30万吨煤炭产能退出任务，房地产去化周期保持在合理区间。国企改革攻坚成效明显，49家驻洛央企、5家市属企业“三供一业”完成分离移交，44家脱钩企业深化改革工作大头落地，27家“僵尸企业”处置阶段性任务顺利完成，市属企业子公司层面9个混改项目成功落地，签约市场化债转股11.9亿元。“放管服”改革力度加大，“一网通办”前提下的“只进一扇门”“最多跑一次”改革全面提速，审批服务事项网上可办率100%、一网通办率95.1%，企业开办时间压缩至4个工作日，新增各类市场主体9.49万户。投融资体制改革不断深化，我市入选全省首批促进科技与金融结合试点地区。农村土地承包经营权颁证基本实现全覆盖。国有林场改革基本完成。政府机构改革顺利实施，政府部门职能进一步优化。开放水平不断提升。自贸区试点任务全面落实，商事制度改革走在全省前列，全省首个交通银行离岸交易中心成功落地，省内首家线下国际贸易“单一窗口”建成投用，洛阳－布哈拉农业综合示范区被评为首批省级境外农业合作示范区。开通洛阳－宁波海铁联运班列。洛阳跨境电商通关平台实现“一站式”通关，跨境电商交易额37亿元。洛阳机场通航城市达26个，全年吞吐量突破130万人次。精准招商成效显著，签约亿元以上重大招商引资项目435个、总投资2319.7亿元，其中10亿元以上项目80个、已开工51个。完成进出口总额143.7亿元，同比增长7.9%。

（四）着力强基础、重统筹，城乡建设协调推进。围绕构建“四级联动”现代城镇体系，大力实施“一中心六组团”发展战略，统筹推进百城建设提质和文明城市创建，全面启动乡村振兴战略，城乡综合承载能力进一步提升。加强基础能力建设。洛吉快速通道黄河大桥、龙门交通枢纽公路客运中心等63项工程相继投用，北环路东段、西环路北段工程竣工在即，“十字加外环”的城市路网格局初步形成。新建城市游园50处，实现了“开窗见绿、出门进园”。打通断头路9条，改造背街小巷50条、老旧小区102个，拆除违章建筑1078处。棚户区改造新开工14449套、基本建成29852套。城市地下综合管廊加快建设。故县水库引水工程全线通水。城市区新增集中供热面积743万平方米，供热普及率达80.1%。生活垃圾综合处理园区建成投用。数字城管平台实现市域一体化运行。500千伏变电站（洛阳东）工程开工建设，220千伏城西、宜阳南等16项电力主网工程建成投用。改善农村基础条件。全面推进农村人居环境整治三年行动，154个建制村环境综合整治如期完成，农村“厕所革命”全面推开。新建标准化卫生室150个，乡、村（社区）综合文化中心3240个。解决农村11.42万人饮水安全问题。前坪水库主体工程进展顺利，小浪底南岸灌区、西霞院灌区列入省十大水利工程。新增有效灌溉面积2万亩、节水灌溉面积2万亩，治理水土流失面积150平方公里。新建农村公路979公里、桥梁1013延米，“四好农村路”建设居全省前列，新安县获评“四好农村路”全国示范县。加大污染防治力度。拆除煤气发生炉5台，完成20台10蒸吨以上燃煤锅炉拆改和提标治理改造。全面建立河长制，“四河三渠”综合治理成效明显，整治河道140公里。全面推进国土绿化提速提质，造林15.6万亩，森林覆盖率达到45.1%，人均公园绿地面积达到11.7平方米，实现了城市“四季常绿、三季有花”。栾川县入选国家“绿水青山就是金山银山”实践创新基地。

（五）着力办实事、增福祉，民生保障持续加强。加快构建公共服务体系，进一步提高民生质量。完成民生支出455.3亿元，占一般公共预算支出的76.2%。市重点民生实事基本完成。扎实推进脱贫攻坚。实施产业扶贫项目1151个，光伏扶贫装机225兆瓦。易地扶贫搬迁工作在全国现场会上受到充分肯定，“十三五”规划安置点全部完工，5.9万贫困群众喜迁新居。积极促进创业就业。返乡创业国家级试点建设扎实推进，发放创业担保贷款23.56亿元，扶持创业近2万人、带动就业6万余人。新增城镇就业11.89万人、再就业2.75万人、农村劳动力转移就业2.81万人。加快发展社会事业。学前教育、高中阶段教育毛入学率分别达到90%、91.5%。新建、改扩建幼儿园76所、农村寄宿制学校114所。职业学校资源整合工作稳步推进，驻洛高校建设迈出新步伐。“书香洛阳”建设取得实效，公共图书馆、城市书房、农家书屋、城市游园服务效能持续提升，15分钟阅读圈、健身圈不断巩固。我市获省十三届运动会第二名。新投用新能源公交车308辆。市第一人民医院、第二中医院恢复公益属性，“互联网+医疗”实现市、县、乡、村全覆盖。持续完善社会保障。提高城乡低保和农村特困人员供养标准。贫困人口基本医疗、基本养老保险实现应保尽保。32万退休人员享受基本养老金调整、增发政策。城乡居民医保门诊特殊病种增加至72个。新建养老床位3013张、农村老年幸福院203个、“儿童之家”150个。维护社会和谐稳定。深入开展“扫黑除恶”专项斗争，依法打击各类违法犯罪行为，群众安全感持续增强。严格落实安全生产责任制，深化重点行业领域专项治理，加强食品药品和特种设备安全监管，安全形势保持平稳。完善社会矛盾排查化解机制，畅通群众诉求渠道，信访总量下降，信访秩序好转，社会大局总体稳定。

（六）着力转作风、提效能，发展环境更加优化。聚焦中央、省委巡视、环保督察及扫黑除恶督导反馈意见整改，扎实开展政府系统转作风提效能专项行动，政府执行力进一步提高。全面贯彻中央八项规定及其实施细则精神，严格执行国务院“约法三章”，驰而不息纠正“四风”，“三公”经费同比下降8.5%。加强法治政府建设，我市连续三年在全省依法行政考核中获得优秀等次，受到省政府通报表彰。自觉接受人大法律监督和工作监督，主动接受政协民主监督，办理人大代表建议293件、政协提案202件。大力加强国防动员、后备力量及人民防空建设，支持军队改革发展，推进军民融合深度发展，持续深化双拥工作，驻洛解放军、武警部队、民兵预备役人员为我市发展作出积极贡献。组建退役军人事务机构，退役军人服务保障工作进一步强化。同时，民族宗教、外事侨务、对台、统计、审计、人防、气象、地震、史志、残疾人工作、妇女儿童事业等都取得了新的成绩。

一年的拼搏奋斗之路，就是一曲昂扬奋进之歌，彰显了全市人民开拓创新、锐意进取的精神风貌，积累的经验弥足珍贵。在实践中，我们深切体会到：必须坚持党的领导，坚定正确政治方向。过去的一年，全市上下在市委领导下，始终向总书记看齐、与党中央对标，瞄准“四高一强一率先”奋斗目标，全面深化“9+2”工作布局，变化有目共睹，成绩令人鼓舞，充分证明市委提出的目标、思路和举措符合中央精神、体现省委要求、紧扣洛阳实际、顺应人民意愿。只要我们基调不变、方向不改、主线不偏、布局不乱，一步一个脚印走下去，洛阳转型升级、高质量发展之路一定会越走越宽广！必须牢记使命担当，保持奋勇争先劲头。“十三五”以来，洛阳被赋予国家区域性中心城市、中原城市群副中心城市、带动河南发展新的增长极等发展定位，迎来了自创区、自贸区、“中国制造2025”等重大机遇，这是中央和省委省政府对洛阳的厚爱与支持，更是全市广大干部群众奋力争先的结果和见证。百舸争流，奋楫者先；千帆竞发，勇进者胜。我们要“跳出洛阳看洛阳，跳出河南看洛阳”，瞄准先进、争创一流，一定能变优势为胜势，化潜力为实力！必须敢于迎难而上，激发拼搏进取斗志。过去的一年，我们顶住了多种压力，经受了多重考验，负重前行，愈战愈勇。面对实体经济之难，我们科学调控、精准施策，经济运行稳中向好；面对结构调整之痛，我们改革创新、转型提质，老工业基地焕发蓬勃活力；面对脱贫任务之重，我们紧盯目标、强力攻坚，决战决胜奠定坚实基础。只要我们不畏艰辛、勇往直前，就没有迈不过的坎、趟不过的河！必须大兴务实作风，弘扬干事创业精神。过去的一年，我们始终把人民对美好生活的向往作为奋斗目标，在百城提质、三大改造、扶贫搬迁、河渠治理等多个领域打造了一批亮点，创造了“洛阳速度”“洛阳品位”“洛阳特色”，得到了上级肯定，赢得了各界赞誉，带给了洛阳人民实实在在的获得感幸福感。只要我们持续发扬“清新简约、务本责实”的工作作风，真抓实干、久久为功，就一定能够保持大好局面，创造更加美好的未来！必须加强团结协作，凝聚各方智慧力量。过去的一年，市委把方向、观大势、谋全局，激励广大党员干部拼搏进取、担当作为，形成了心齐气顺、风正劲足的良好氛围。各级各部门担当尽责、创先争优，各垂直部门胸怀洛阳、服务大局，工商界、科技界人士开拓创新、拼搏实干。广大城市建设者昼夜奋战、克难攻坚，打造了一个个深得民心、令人赞叹的精品工程。广大农民群众投身广阔天地，推动乡村振兴。特别是奋战在基层一线的同志们，勇挑重担、任劳任怨，为推进脱贫攻坚、污染防治、安全维稳、防范风险作出了突出贡献。各行各业、各条战线的劳动者都在为洛阳发展加油助力、增光添彩，同心协力描绘出洛阳在中原更加出彩中的华丽篇章！

各位代表，奋斗铸就辉煌，实干成就梦想！过去一年的成绩来之不易，主要得益于习近平新时代中国特色社会主义思想的科学指引，得益于省委、省政府对洛阳的关怀厚爱，得益于市委的正确领导与科学决策，得益于市人大、市政协的有效监督和全力支持，离不开中央、省、市各大媒体的大力支持和省驻洛各单位的共同参与，记载着历届政府班子打下的坚实基础，凝聚着全市人民的无私奉献和社会各界的帮助支持。在此，我代表市人民政府，向710万洛阳人民，向人大代表、政协委员，向各民主党派、人民团体，向离退休老领导、老同志，向驻洛部队、武警官兵和公安干警，向所有关心支持洛阳发展的社会各界和海内外朋友，表示衷心的感谢并致以崇高的敬意！

肯定成绩的同时，我们也清醒地认识到，我市发展还面临着诸多困难和问题：一是结构调整任务艰巨。传统产业占比仍然较大，新兴产业发展快但总量偏小，现代服务业发展相对滞后，实体经济面临较大困难，质量效益需要持续提升。二是创新发展动力不足。高层次创新人才缺乏，创新资源协同作用发挥不充分，一些制约创新活力的体制机制障碍仍然存在，科技创新对经济发展的带动能力有待提高。三是民生事业欠账较多。脱贫攻坚任务艰巨，住房、教育、医疗、就业、养老、生态环保等方面还存在不少短板。四是风险隐患不容忽视。企业发展、金融运行、安全生产等领域的风险隐患仍然较多，推进社会治理创新、维护社会稳定的任务还很艰巨。五是职能转变还不到位。作风建设仍需持续深化，政府效能和服务水平有待进一步提升。对此，我们一定高度重视，认真加以改进，全力做好工作，决不辜负党和人民的重托！

二、2019年总体工作要求

今年是新中国成立70周年，是全面建成小康社会的冲刺之年，也是我们全面深化“9+2”工作布局、奋力实现“四高一强一率先”奋斗目标的关键之年。市委十一届九次全会描绘了推进转型发展、高质量发展、谱写浓墨重彩洛阳篇章的美好蓝图，这是洛阳人民共同的梦想。新的一年，我们要在追梦路上阔步前行、接续奋斗，努力抓住新机遇、塑造新形象、展现新作为。

今年政府工作的总体要求是：以习近平新时代中国特色社会主义思想为指导，全面贯彻党的十九大和十九届二中、三中全会精神，认真落实中央经济工作会议、省委十届八次全体（扩大）会议、市委十一届九次全体（扩大）会议精神，统筹推进“五位一体”总体布局，协调推进“四个全面”战略布局，坚持稳中求进工作总基调，坚持新发展理念，坚持推动转型发展、高质量发展，坚持以供给侧结构性改革为主线，坚持深化市场化改革、扩大高水平开放，贯彻巩固、增强、提升、畅通的方针，坚决打好“三大攻坚战”，持续推进“四个着力”、打好“四张牌”，全面深化“9+2”工作布局，统筹推进稳增长、促改革、调结构、惠民生、防风险工作，着力推进产业转型、动能转型、城市转型、生态转型、社会治理转型、体制机制转型，使经济运行保持在合理区间、发展质量效益不断提升、特色优势日益厚植，增强人民群众获得感、幸福感、安全感，保持经济持续健康发展和社会大局稳定，为全面建成小康社会收官打下决定性基础，以优异成绩庆祝中华人民共和国成立70周年。

今年全市经济社会发展的主要预期目标是：生产总值增长8%－8.5%，一般公共预算收入增长7.6%，规模以上工业增加值增长8%，固定资产投资增长9%，社会消费品零售总额增长10.5%，主要经济指标增速高于全省平均水平。在创新发展方面，研究与试验发展经费投入强度达到2.3%，战略性新兴产业、高新技术产业增加值占规模以上工业比重分别达到20%和40%，科技进步对经济增长的贡献率达到63%以上。在协调发展方面，常住人口城镇化率达到59.2%左右，农民人均可支配收入增速高于城镇居民收入增速，人均预期寿命持续提高，教育、医疗、文化、体育等社会事业全面进步。在绿色发展方面，单位生产总值能耗下降幅度、主要污染物排放量完成省下达目标。在开放发展方面，进出口总额保持稳定增长，营商环境在全省评价排名位居前列。在共享发展方面，居民人均可支配收入增速高于GDP增速，汝阳、嵩县脱贫摘帽，5.4万农村贫困人口稳定脱贫，城镇新增就业9万人以上，城镇调查失业率和登记失业率分别控制在5.5%和4.5%以内，居民消费价格指数控制在3%左右。

三、2019年重点工作

实现今年预期目标，关键是要抓住重点、保持定力、精准发力，持续巩固转型发展、高质量发展的态势。

（一）综合施策稳定运行，巩固经济稳中向好态势。认真贯彻巩固、增强、提升、畅通八字方针，聚焦“六稳”目标，积极扩大需求，加强综合调控，力促经济持续平稳增长。一是狠抓项目增投资。全力实施“97172”合理有效投资行动计划，扎实推进9大体系、近70个重大专项、1138个总投资7113亿元、年度计划投资2258亿元的重大项目，确保455个省市重点项目全年完成投资1387亿元。开工建设万基控股10万吨高精度铸轧卷、中钢耐火绿色化改造等521个项目，加快大鱼沟抽水蓄能电站、格力智能制造产业园等617个续建项目进度，确保新星轻合金材料产业园、北玻硅巢材料等400个项目年内竣工。力争完成洛阳石化炼油扩能改造项目一期和日照－濮阳－洛阳原油管道主体工程，开工洛阳－新郑机场航空煤油管道。加强项目谋划衔接，积极对接全省“7819”扩大有效投资专项行动，争取更多项目进入国家重大建设项目库和省计划盘子。二是扩大内需促消费。出台促进消费三年行动计划和扩容提质措施，利用重大节日、节会扩大输入型消费，增强文化、旅游消费带动作用，培育健康、教育、医疗、养老、家政等新兴消费增长点，进一步巩固“消费超投资”的态势，积极打造区域性消费中心城市。落实网络提速降费政策，加快信息消费试点城市建设，推动互联网与传统消费渗透融合。支持中浩德等电商物流产业园发展跨境电商业务，在自贸区布局若干保税体验店。积极推动汝阳、洛宁、嵩县等国家级电子商务进农村综合示范县建设，培育发展一批各具特色的“淘宝镇”“淘宝村”。创建10个以上A级乡村旅游景区，因地制宜发展特色民宿、田园综合体、乡村创客基地等乡村旅游消费产品。优化供地结构，简化审批流程，加快房地产项目建设，有序释放刚性需求和改善性需求。加强消费领域基础设施建设，营造安全放心的消费环境，让老百姓能消费、愿消费、敢消费。三是精准服务稳企业。深化地企合作，拓展领域空间，加强对接服务，抓好项目落地，推动与中信集团、国机集团、格力电器等企业合作取得更大成效。认真落实全省“千名专家进百县帮万企”服务活动，指导帮助企业改造升级、绿色发展。落实更大规模减税降费政策，对小微企业和科技型初创企业实施普惠性税收减免，全面实施收费项目清单制，力争全年降低企业成本30亿元以上。开展重点领域金融服务专项行动，设立融资担保基金，扩大还贷周转金资金池规模，解决中小微企业融资难、融资贵问题，增强金融服务实体经济的能力。四是扶持民企增活力。建立“亲”“清”新型政商关系，开展促进民间投资健康发展专项行动，完善落实激励政策，遴选培育一批大型民营骨干企业，引导支持民营企业走“专精特新”发展之路。建立向民间投资推介项目长效机制，优先支持民间投资的重大项目纳入省市重点项目盘子。继续实施“小巨人”企业培育工程，全年新增“小巨人”企业10家、“小升规”企业超百家。设立中小企业发展专项资金，健全中小企业公共服务体系，力争全年省级以上平台服务中小企业3000家（次）。五是防范风险守底线。强化底线思维，密切关注非法集资、问题楼盘、企业运行等重点领域的风险隐患，建立健全风险研判、决策评估、防控责任机制，完善预案，精准施策，着力解决可能影响高质量发展的突出问题，坚决守住不发生系统性区域性风险的底线。高度关注房地产市场运行态势，促进房地产市场长期平稳健康发展。

（二）着力推动制造业高质量发展，带动产业转型升级。全面深化“565”现代产业体系，把推进制造业高质量发展放在更加突出的位置，坚持“四双联动”，实施“四大行动”，持续提升产业链水平，加快建设全国重要的先进制造业基地、现代服务业基地。一是做大做强先进制造业。深入开展智能制造引领、重点技术攻坚、传统产业提质、新兴产业培育“四大行动”，推动重点企业提质倍增，引导企业增品种、提品质、创品牌，积极推进格力中央空调及智能制造等重大项目建设，力争机器人及智能装备产业主营业务收入增速超20%。抓住省政府重点支持主营业务收入100亿元以上重大制造业企业和50亿元以上重点制造业企业的机遇，推动我市制造业企业做大做强。认真筹备第四届洛阳国际机器人暨智能装备展览会。二是加大传统产业改造升级力度。支持500家规上工业企业实施“三大改造”，抓好276个重点改造项目，全年完成投资291亿元以上。突出智能化改造，推广应用1500台以上机器人及智能装备，培育4个省级智能工厂、8个智能车间。深入开展绿色制造体系建设，培育4家绿色工厂，实现重点高耗能行业节能监察全覆盖。大规模实施“五十百”技改提升工程，确保全市技改投资占工业投资比重过半，争取中科院智能农机工程实验室落户洛阳，推动省智能农机创新中心升格为国家级创新中心。开工建设忠旺集团100万吨铝精深加工项目。积极对接推进PX和PTA项目前期工作，争取百万吨大乙烯项目纳入中石化集团发展盘子和国家石化产业“十四五”发展规划，实现全产业链发展，打造“一基地两园区”国家级高端石化产业集群。三是培育壮大新产业新业态新模式。认真落实全省10个重点培育新兴产业发展行动，大力发展大数据、人工智能等新兴产业，支持发展共享经济、平台经济，加快工业互联网平台建设，抓好工业互联网标识解析体系二级节点建设，推动900家工业企业上云。培育一批服务型制造示范企业（平台），完成“两化”融合贯标企业8家、对标80家。积极推动电信普遍服务试点项目建设，确保4G网络实现全覆盖，抓好5G网络试点。四是推动重点产业园区提速提效。完善六大产业园基础设施，强化上下游延伸、左右链配套、集群式发展，积极推进银隆新能源汽车产业园整车及电池、中航光电技术产业基地二期、大数据产业园、景安IDC云计算产业园二期等项目建设，培育一批高端产业集群。深入实施产业集聚区高质量发展专项行动，推动产业集聚区“二次创业”。五是提高现代服务业发展水平。加大“引金入洛”力度，持续推进多层次资本市场建设，不断扩大金融市场供给，争取引进2－3家金融机构，全市资本市场融资达到285亿元。积极推进现代物流业发展，完成顺丰创新产业园前期工作，加快建设万邦国际集团进出口贸易中心、乾发供应链跨境电商项目，建成投运华晟物流园铁路集装箱作业区、中储综合物流产业园铁路专用线。积极发展冷链物流，建设冷库6万立方米以上。加快发展科技服务业，启动建设洛阳市科技创新综合服务平台。推进电子商务转型升级行动计划，创建一批省级电子商务示范基地和示范企业。推动洛阳有色金属交易中心尽快验收运营。争创国家服务外包示范城市。

（三）强化创新驱动发展，实现动能转型新突破。坚持“打造一个龙头、强化一个支撑、抓好三大举措、实现一个带动”，全面深化现代创新体系，促进“政产学研金用介”各类要素高效配置，加快建设国家创新型城市。一是强化创新龙头带动。全面启动高新区体制机制改革，稳步推进自创区扩点带面，有序复制推广先行先试政策，促进“三区”融合发展。围绕先进装备制造、新材料、增材制造等领域，积极引进国内外知名大学、科研院所与驻洛高校院所合作共建一批新型研发机构。二是加快创新资源集聚。聚焦培育“四个一批”，持续实施“双倍增”行动，新培育高新技术企业100家，新备案科技型中小企业800家，重点扶持20家创新龙头企业做大做强；新建创新平台（载体）300个，支持863创智广场等申建国家级科技企业孵化器，鼓励引导清华洛阳基地等申建省级创新平台，建设提升河南科技大学科技园等15个创新创业孵化载体。支持一拖集团、普莱柯公司申建国家级创新中心，持续提升现有国家、省、市级创新中心和重点实验室建设水平。依托各类资源，培育建设高水平智库。三是壮大创新人才队伍。认真落实“1+4”人才政策，推动第三批“河洛英才计划”团队及项目加快落地，启动实施第四批项目，持续推进“河洛工匠计划”，办好“2019科技助力民营经济高质量发展院士洛阳行”、2019中国（河南）直通硅谷创新创业大赛、第四届清华校友三创大赛先进制造行业总决赛，积极支持科学家工作室建设，大力引进高校毕业生和高层次、高技能、实用型等各类人才，让河洛大地成为英才荟萃、大显身手的广阔舞台。四是推动创新成果转化。积极对接省“十百千”转型升级创新专项，重点实施工业CT、高铁轴承产业化项目，抓好智能农机装备、电动重卡、重大动物疾病防控等关键技术攻关。加快省促进科技与金融结合试点地区建设，完善科技金融政策扶持体系，提高天使投资基金、创投基金、种子基金运营效率，力争我市企业在“科创板”上市突破。组建省级产业技术创新战略联盟4－6个，培育市级以上技术转移示范机构8家，启动运营科技中介超市，科技金融大市场全年挂牌项目800个以上，技术合同成交额突破40亿元。五是深化军民融合创新。建立完善军民融合创新推进机制，积极申建国家军民融合创新示范区，高水平运营500亿元省军民融合产业投资基金。加快推进军民融合产业园建设，支持中信重工建设洛阳先进装备及军民融合产业创新科技园，攻克一批关键核心技术“卡脖子”问题。

（四）持续全面深化改革，积极破解体制机制转型障碍。坚持“四个聚焦”“四个围绕”，大力推进重点领域和关键环节改革，进一步释放微观主体活力，全面深化现代市场体系。一是巩固供给侧结构性改革成果。综合运用市场化、法治化手段，坚定不移淘汰落后产能、化解过剩产能，严格控制钢铁、水泥、电解铝等行业新增产能，关停高耗能高污染高排放企业。确保6月底前完成剩余9家“僵尸企业”破产终结，做好债务化解、资产处置等后续工作。谋划实施一批重点项目，下大力气补齐基础设施和公共服务等方面的短板。二是持续深化国资国企改革。打好国企改革攻坚“扫荡战”，深化产权、组织、治理结构改革，积极稳妥推进洛轴混合所有制改革，推动中铝洛铜、中钢洛耐通过搬迁改造、内部改革实现转型发展，国宏集团、城投集团、旅发集团等企业向国有资本投资（运营）公司转型，推动市属企业内部重组整合，强化集团公司职能改革，年底前争取完成5户市属企业子公司混改。加快推进新排查企业的脱钩改制，确保“改彻底、改规范”。进一步规范国资监管，抓好改（组）建国有资本投资（运营）公司试点工作。三是下大力气优化营商环境。持续推进“放管服”改革，深化商事制度改革，实行“容缺办理”“多评合一”“联合审验”，启动营商环境预评价工作，打造一流营商环境。抓好“一网通办”前提下的“只进一扇门”“最多跑一次”改革，持续开展“减证便民”行动，全面落实“三级十同”，加快市行政服务平台和市政务云平台建设，推动政务信息整合和数据共享，促进“互联网+政务服务”深度融合，稳定实现“网上可办”“一网通办”两个100%，确保投资项目全流程审批时间压减至92个工作日以内。加强市场综合监管，推广部门联合“双随机、一公开”监管模式。四是统筹抓好其他重点改革。加快现代医院管理制度建设，稳步推进医药卫生体制改革，大力支持社会办医。逐步推进财税体制、投融资体制改革。深化公交集团改革。3月底前基本完成政府机构改革。

（五）推动全方位高水平开放，打造中西部对外开放新高地。全面深化现代开放体系，实行更加积极主动的开放战略，加大力度引进来、积极稳妥走出去，提高开放型经济发展水平。一是高水平建设自贸区。认真落实国家153项经验、省160项试点任务和洛阳片区200项试点任务，积极开展首创性、差异化的改革探索。加快基础设施建设，确保9月底前自贸大厦具备使用条件、综合保税区具备封关运营能力。加大龙头型、“独角兽”型产业项目引进力度，力争自贸区新入驻企业2000家，集聚国内外行业龙头企业10家以上。二是提升开放平台优势。高标准建设口岸、跨境电商、外贸基地等各类开放平台，加快发展公铁集疏、多式联运，推动铁路国际货运通道“东联西进”常态化、高频次运行，推进一拖编组站改造为陆港多式联运物流中心，谋划指定口岸建设和机场三期改扩建工程，推进一类航空口岸扩大开放。加快国际贸易“单一窗口”建设，提升贸易便利化水平。三是大力开展精准招商。围绕构建“565”现代产业体系，紧盯国内外500强、行业50强，进一步拓宽开放领域，推动精准招商提速提质，力争全年签约10亿元以上项目80个。深化与格力电器、兵器集团、中国节能等企业集团合作，推进一批合作项目落地实施。四是扩大对外经贸合作。发挥“一带一路”主要节点城市优势，推动出口市场多元化，扩大进口规模，实现外贸进出口稳中提质。巩固扩大中信重工、一拖集团、洛钼集团、伊电集团等优势企业“走出去”成果，加快洛阳－布哈拉农业综合示范区建设，鼓励支持有实力的企业扩大国际产能合作和经贸合作。积极参加广交会、进博会、东盟博览会等国际贸易活动，高质量办好第十三届中国（河南）国际投资贸易洽谈会洛阳分会场活动。

（六）全面提升城镇综合承载能力，筑牢城市转型基础支撑。认真落实全省百城建设提质工程暨文明城市创建工作推进会精神，全面深化现代城镇体系、现代基础设施体系，持续实施“一中心六组团”城市发展战略，统筹做好“规划、建设、管理”三篇文章，把城市建设的“工笔画”越画越漂亮，让城市管理的“交响曲”越奏越和谐！一是加快推进副中心城市建设。主动向上对接，抓紧启动编制洛阳都市圈规划，推进洛济、洛汝、洛渑、洛巩等产业带规划建设，积极谋划一批跨区域合作项目，推动洛阳与豫西北城市联动发展，增强对陕东、晋南地区的资源聚合和区域辐射作用。完成城市五期总规修编和国土空间规划，把既定发展定位落到实处。二是全面铺开百城提质工程。坚持“四条路径”一起发力，“五项治理”一起推进，“四个品质”一起提升，促进中心城区和县城由外延扩张向内涵提质转变。突出抓好今年确定的中心城区和各县（市）城建项目，全力推进滨河南路东延、开元大道西延等97项路桥工程，确保牡丹文化节前王城大道快速路洛河以南段、北环路东段等主线通车；5月底前瀍涧大道、北环路西段竣工通车，“十一”前王城大道快速路洛河以北至机场路段、南昌路改造、洛吉快速、洛新快速等实现通车，开工建设洛伊、洛偃快速路。打通27条断头路。新建、改扩建中心城区小游园50个。加快地下综合管廊建设，实施一批供水节水、排水防涝、热力燃气、污水垃圾处理等补短板项目。新建停车场52个，新增停车位1.55万个以上。在中心城区实施32个老旧小区、50条背街小巷、15个农贸市场功能改造提升和8个市场疏解外迁，建设提升关林、洛邑古城、广州市场等步行街。三是加快综合交通体系建设。围绕建设国家重要交通枢纽，加快实施一批重大交通基础设施项目，积极推进呼南高铁豫西通道规划研究工作，加快推进轨道交通、组团快速通道、动车组存车场、三洋铁路洛阳段建设。积极推进洛济、尧栾西等高速公路建设，迅速掀起二广高速公路城区段改扩建和新伊、栾卢、渑栾高速施工高潮。开展组团高速东南环线等项目前期工作，争取列入省高速公路路网规划。加快高铁龙门站综合交通枢纽北广场、洛阳火车站综合交通枢纽、谷水综合交通枢纽、万安山通用航空机场建设，推动栾川、洛宁通用航空机场前期工作。四是提高城市精细管理水平。持续推进交通秩序“七策治堵”、生态环境“三力治污”、市容卫生“四化治脏”、城乡建设“四抓治违”、公共服务“三字治差”，扎实开展文明城市创建，抓好新时代文明实践中心试点建设，协同开展村镇、单位、家庭、校园等群众性精神文明创建活动。提高网络治理能力，打造清朗网络空间。五是增强县域经济支撑能力。全面贯彻习近平总书记县域治理“三起来”的重要指示，着力补短板、破瓶颈，保障土地、人才等要素供给，激发活力、释放潜能，推动县域经济高质量发展。

（七）大力实施乡村振兴战略，加快推进农业农村现代化。认真落实中央、省委农村工作会议和全省扶贫开发工作会议精神，以“五个振兴”为路径，加快建设产业兴旺、生态宜居、乡风文明、治理有效、生活富裕的新农村。一是决战决胜脱贫攻坚。持续深化“一转三推进”，统筹抓好“六个精准”“五个一批”，全面推进14个脱贫攻坚重大专项，突出抓好产业扶贫攻坚，加大基础设施和公共服务设施建设力度，完成易地扶贫搬迁集中安置点配套设施建设，精准落实因病因残致贫、孤寡老人等特殊贫困群体的保障措施，高质量完成嵩县、汝阳脱贫摘帽、5.4万贫困人口脱贫任务，确保整体脱贫、稳定脱贫。二是培育发展新型农业经营主体。完善农业产业化龙头企业服务保障机制，力争年内重点龙头企业达到400家。新培育产业化联合体15个，支持农业专业合作社、家庭农场产业化发展。大力发展农村集体经济，采取使用权入股、租赁、自主经营等形式，增加村集体经济收入，力争全市80%以上的行政村有集体经济收入，其中80%以上超过5万元。三是深入推进农业供给侧结构性改革。坚持质量兴农、绿色兴农、品牌强农，积极推进“互联网+现代农业”，促进农村一二三产融合发展。严格落实粮食安全责任制，粮食总产稳定在40亿斤以上。坚持“四优四化”，大力调整农业结构，压缩旱地玉米种植面积，发展优质强筋小麦25万亩，增加设施蔬菜1500亩，扩大杂粮种植面积。新发展沟域经济示范区10个，新认证无公害农产品40个、绿色食品15个、地理标志农产品2个。落实农机购置补贴政策，提高农业机械化水平。四是完善农村基础设施。实施289个行政村饮水安全巩固提升工程。确保前坪水库年内建成并下闸蓄水，小浪底南岸灌区3月份开工，西霞院灌区年内开工，争取小浪底水库库周地质灾害处理获国家立项。加快推进交通领域“千组万户通达工程”“村村通客车五化提质工程”，建设800公里“四好农村路”。实施新一轮农村电网改造升级。加快构建覆盖县、乡、村的农村物流网络体系。五是改善农村人居环境。学习浙江“千万工程”经验，开展“百村示范、千村整治”，推进村庄垃圾污水整治、农村“厕所革命”，绿化美化村庄300个，改造卫生厕所24万户，确保汝阳、嵩县、伊川、洛宁、偃师5县（市）农村生活垃圾治理通过省级验收，让农村净起来、绿起来、美起来。

（八）加大污染防治和生态建设力度，实现生态转型更大成效。深入贯彻习近平生态文明思想，全面深化生态环境建设体系，扎实推进“五大行动”，抓好中央环保督察“回头看”和省委省政府环保督察问题整改，让洛阳的天更蓝、山更绿、水更清。一是强力实施大气污染防治。积极推进汾渭平原区域综合治理，抓好国家清洁取暖试点城市建设，严格落实“六控”措施和建设工地“七个百分之百”要求，加强“散乱污”企业整治，取缔燃煤散烧设施，统筹做好“燃煤双代”，推进中心城区煤电机组基本清零，解决好结构性、季节性、区域性污染问题，确保全市PM10、PM2.5年均浓度和优良天数完成省定目标。新投用600辆清洁能源公交车。加快推进“引热入洛”工程，确保中心城区新增集中供热面积400万平方米，集中供热率、清洁取暖率分别达到85%和95%。二是持续深化“四河三渠”综合治理。推动河长巡河常态化。加快推进伊河下游段4.5公里、洛河下游段5公里、瀍河洛孟路至孟津段6.9公里、涧河衡山路至新安界15.2公里、伊洛瀍涧县域段63公里重点河道，以及铁路防洪渠、大明渠、中州渠综合治理，上半年实现瀍河、涧河两岸沿线截污管道全覆盖，提高新区水系管理水平。巩固黑臭水体整治成果，开展水源地污染综合防治，确保7个地表水国、省重点监控断面达标率100%。三是大力推进国土绿化提速提质。以“11555”绿化工程为统揽，坚持“规划见绿、见缝插绿、提质优绿、协力植绿”，高标准实施“七绿”提速提质工程，抓好国家储备林基地项目建设，迅速掀起春季植树造林热潮，完成19万亩造林绿化和75.3万亩森林抚育改造任务。全面提升4个森林公园，培育5个森林特色小镇、70个森林乡村、3个森林康养基地。四是积极实施生态修复行动。对接省沿黄生态带规划建设，加快吉利区黄河湿地建设，抓好嵩县陆浑湖和伊川伊河国家湿地公园试点工作，在中心城区伊洛瀍涧四河上建成甘泉河等6个湿地。扎实推进土壤污染综合防治先行区建设，抓好栾川、嵩县、汝阳、洛宁治理修复项目，加强重金属污染、农业面源污染防治和固体废物处理。

（九）加快建设国际文化旅游名城，进一步提高文化软实力。全面深化文化传承创新体系，深入挖掘历史文化资源，积极推进文化旅游“三个转变”，延续城市文脉，彰显古都特色。一是加强文化遗产保护利用。主动融入国家“大运河文化带”战略，深入推进华夏历史文明传承创新区建设，突出抓好隋唐洛阳城国家历史文化公园、老城历史文化街区二期、释源大道等重点项目，实施天街北延和里坊区环境整治，启动东城墙遗址保护展示工程前期工作，确保九洲池一期牡丹文化节前对外开放、南城墙年内全线完工，尽快实现宫城区由景点到景区连片发展，逐步勾勒隋唐洛阳城的历史轴线和47平方公里的都城轮廓。二是加快建设“博物馆之都”。依托重要文化遗产资源，打造一批骨干博物馆，推进牡丹博物馆、工业遗产博物馆、客家文化博物馆等建设，确保隋唐大运河遗址博物馆年内开工，二里头遗址博物馆及考古遗址公园10月份对外开放，班超纪念馆等年内建成开放。积极推进伊川、汝阳、嵩县等一批县域博物馆建设。创新博物馆运营机制，加快数字化博物馆建设，提升综合功能和服务水平，开发博物馆旅游精品线路，推动文博产业发展。三是促进文化旅游融合发展。强化龙门石窟等重点景区龙头带动作用，支持栾川、嵩县、孟津、洛龙等县（区）创建国家全域旅游示范区，加快十大文化产业园建设，培育文化旅游新业态，打造旅游目的地品牌。谋划建设洛阳国际会展中心，精心办好第37届中国洛阳牡丹文化节、中国－中东欧国家文化遗产论坛、第二届世界古都论坛、2019河洛文化旅游节，力争全年接待游客1.4亿人次以上、增长7%左右，旅游总收入1300亿元以上、增长15%左右。

（十）切实保障和改善民生，开创社会治理转型新局面。坚持以人民为中心的发展思想，全面深化公共服务体系，集中更多资源投向民生领域，认真办好51项86件民生实事，有诺必践、取信于民，推动“七个有所”实现新突破，让全市人民拥有更多获得感、幸福感、安全感。一是更加重视就业创业。积极推进公共就业服务专项行动，抓好高校毕业生、退役军人等重点群体就业，稳定农村劳动力转移就业，支持农民工等群体返乡下乡创业。发放创业担保贷款15亿元，完成失业人员就业技能培训1.8万人、农村劳动力职业技能培训2.5万人，新增城镇就业9.86万人，就业困难人员实现就业6000人。力争全市省级创业孵化示范基地达到8家。二是推动教育优先发展。坚持立德树人，大力发展素质教育，进一步提高教育教学质量。优化中小学和幼儿园规划布局，发展普惠性学前教育，新建、改扩建50所公办幼儿园，新增6000个幼儿学位；新建、改扩建城市区义务教育学校18所、农村义务教育寄宿制学校56所。招聘特岗教师600名，培养农村小学全科教师免费师范生260名。加快推进全国青少年校园足球改革试验区建设。开工建设市委党校新校区、河南中医药大学洛阳平乐正骨学院。加快洛阳职业技术学院新校区建设，力争9月份新生入住。支持驻洛高校建设一流学科。三是加强卫生健康文化服务。围绕健康洛阳建设，持续深化综合医改，开工建设省第二儿童医院（市儿童医院）、伊滨医院，加快洛阳中医院伊滨医院建设。扎实推进健康扶贫，加强贫困地区医疗机构标准化建设，下沉医疗力量，降低医疗成本。加快县（市）人民医院提质升级，改造提升村卫生室200个。深入开展“互联网+医疗健康”便民惠民活动，解决群众看病难、看病远问题。启动洛阳市奥林匹克中心建设，确保文博体育公园二期上半年建成投用。争创国家全民运动健身模范城市，认真筹办省第十四届运动会，积极承办高端体育赛事。深化提升国家公共文化服务体系示范区、国家文化消费试点城市建设，积极申报国家文化生态保护区。持续建设“书香洛阳”，实施城市书房服务提升工程，新建城市书房50座。四是提高社会保障水平。加强社会救助体系建设，提高城乡低保和特困人员供养标准。提升改造农村敬老院，推进市、县政府示范性养老机构建设，新建农村老年幸福院200个，新建社区养老服务中心20个，新建养老床位2000张，加快“智慧养老”平台建设，抓好居家和社区养老服务改革试点工作。完善住房市场体系，积极培育和发展住房租赁市场，探索共有产权住房保障模式，加强公租房建设与管理，完成分配入住3000套。建设市级统建青年人才公寓900套。提高城乡居民基本养老保险基础养老金最低标准。对80岁以上参加城乡医保的高龄老人在政策范围内的住院费用，报销比例再提高5个百分点。五是推动社会治理创新。持续深入开展“扫黑除恶”专项斗争，做好中央督导组反馈问题整改，依法严厉打击违法犯罪活动。加快应急管理体制改革，加强应急救援体系建设，提高抗御风险和防灾减灾能力。积极创建国家安全发展示范城市，严格落实安全生产责任制，加强隐患排查治理和风险防控，坚决防范各类事故发生，确保实现“一杜绝、三下降”，全面提升城市安全发展水平。加强信用体系建设，强化食品药品安全监管，让人民群众吃得更安全、用药更放心。学习“枫桥经验”，创新工作方法，依法妥善解决群众合理诉求，提升信访工作法治化水平，确保社会和谐稳定。深入开展宪法学习宣传和“七五”普法教育。加强全民国防教育，巩固提升国防动员和后备力量建设成果，继续支持国防、军队调整改革和驻洛部队建设，完善退役军人服务保障体系，深入开展双拥共建工作，落实各项优抚政策，争创全国双拥模范城八连冠。

四、建设人民满意的服务型政府

新形势、新任务对政府自身建设提出了新目标、新要求。我们将始终坚持党的领导，坚持以人民为中心，牢记政府职责使命，全面提升履职能力，高水平做好政府各项工作，让人民群众更放心、更满意、更信赖！

旗帜鲜明讲政治，始终对党绝对忠诚。自觉以习近平新时代中国特色社会主义思想为引领，进一步树牢“四个意识”，坚定“四个自信”，坚决做到“两个维护”，听从总书记号令，服从党中央指挥，严守政治纪律和政治规矩，全面加强政府系统意识形态工作，牢牢把握正确的政治方向，确保党中央国务院、省委省政府和市委的各项决策部署落到实处、见到实效。

严格规范重法治，大力推进依法行政。将政府工作纳入法治化轨道，全面落实政府法律顾问制度，加强重大行政决策、规范性文件、重大行政执法决定合法性审查，深入调查研究，提高政府决策科学化、民主化、法治化水平。做好政府立法工作，加强行政复议规范化建设。全面推行行政执法公示、执法全过程记录和重大执法决定法制审核制度，推进行政执法严格、规范、公正、文明。加大政务公开力度，严格执行市人大及其常委会的决议决定，主动接受人大、政协监督，自觉接受监察监督和司法监督，虚心接受社会和舆论监督，加强审计监督，让权力在阳光下运行。

持之以恒提效能，加快转变政府职能。推进政府机构改革，持续优化机构设置和职能配置，推动政府治理能力再上新台阶。紧盯群众办事、企业发展和政务服务中的堵点、痛点、难点，持续深化“放管服”改革，打通政策落实的“最后一公里”，破除一切不利于发展的桎梏，激活一切有利于创新的因子，为企业松绑、为创新清障、为公平护航，让百姓省心省力，打造宜业宜居、近悦远来的发展环境。

主动作为敢担当，持续改进工作作风。落实正向激励和容错纠错机制，大力弘扬焦裕禄精神、红旗渠精神、愚公移山精神、右玉精神，勇于担当尽责，敢于克难攻坚。持续倡树“清新简约、务本责实”的作风导向，按照“钉钉子”“透竹竿”“剥洋葱”的要求，狠抓工作落实，加强跟踪问效，推动各项决策落地生根、开花结果。驰而不息纠正“四风”，严肃整治懒政怠政。践行“一线工作法”，保持雷厉风行的作风，说了就办、定了就干、干就干好。

筑牢底线守规矩，全面加强廉政建设。坚决扛起全面从严治党主体责任，扎实推动政府系统党风廉政建设和反腐败工作向纵深发展，全面净化政府系统政治生态。严格执行中央八项规定及其实施细则精神，认真落实中央、省、市各项纪律要求，严控“三公”经费，带头过紧日子，厉行节约，反对浪费。加强纪律教育和纪律执行，让政府系统广大公务人员知敬畏、存戒惧、守底线，永葆为民务实清廉的公仆本色。

各位代表，追逐梦想勇担当，砥砺奋进再起航。让我们更加紧密地团结在以习近平同志为核心的党中央周围，在省委省政府和市委的坚强领导下，乘势而上、奋勇向前，全力推动洛阳转型发展、高质量发展，在中原更加出彩中奋力谱写浓墨重彩的洛阳篇章，为全面建成小康社会收官打下决定性基础，以优异成绩向新中国成立70周年献礼！
